# Bulletin of North Carolina Department of Agriculture

**Published:** 1887-02

**Authors:** 


The Bulletin of the North Carolina Department of Agriculture for November is before us. It contains a detailed report of the proceedings of the "Convention of Northern Settlers in North Carolina."

The object of the Convention is to induce immigration to North Carolina. A creditable showing of the advantages of residence in this State is set forth in the proceedings.

Possibly this State would be a good place for homœopathic doctors to locate. At all events, information can be obtained of John T. Patrick, General Agent, North Carolina Immigration Bureau, Raleigh, North Carolina.

